# Editorial: Small vessel disease: From diagnosis to organized management pathways

**DOI:** 10.3389/fneur.2022.1120426

**Published:** 2023-01-10

**Authors:** Marialuisa Zedde, Jennifer Linn, Aristeidis H. Katsanos, Rosario Pascarella

**Affiliations:** ^1^Neurology Unit, Stroke Unit, Azienda Unità Sanitaria Locale-IRCCS di Reggio Emilia, Reggio Emilia, Italy; ^2^Institute of Diagnostic and Interventional Neuroradiology, University Hospital Carl Gustav Carus, Dresden, Germany; ^3^Division of Neurology, McMaster University and Population Health Research Institute, Hamilton, ON, Canada; ^4^Neuroradiology Unit, Azienda Unità Sanitaria Locale-IRCCS di Reggio Emilia, Reggio Emilia, Italy

**Keywords:** small vessel disease (SVD), cerebral microbleed (CMB), arterial hypertension, diabetes, inflammation, WMH

Cerebral small vessel disease (SVD) is a major underlying cause not only of ischemic and hemorrhagic stroke but also of cognitive impairment and dementia (1). It includes several different diseases, both sporadic [e.g., arteriolosclerosis (type I SVD) and cerebral amyloid angiopathy (type II SVD) (2)] and inherited or genetic. Globally, the standardized definition of SVD is based on neuroimaging markers, with variable specificity for the different forms of disease as opposed to mixed SVD (3, 4). The prevalence of SVD makes it a crucial public health concern for health care providers and politicians, and it is of paramount importance that the broader medical community has a good understanding and awareness of SVD for the prevention of stroke and cognitive impairment (5) and for the provision of tailored treatment (6), in particular in the comorbid patient and in the organization of pathways of care in the near future. Indeed, SVD has a major impact as a comorbidity in patients with stroke arising from other defined causes (7); additionally, it increases hemorrhagic risk and worsens functional outcomes in patients with stroke (8) in general and in patients treated with intravenous thrombolysis and/or endovascular thrombectomy (9). Neuroimaging markers of SVD are also predictive of intracranial bleeding risk in patients with cardioembolic stroke undergoing anticoagulant treatment (10).

For these reasons, SVD has been attracting increasing amounts of attention in recent years on various levels, from preclinical and clinical research on disease mechanisms to its phenotyping and the standardization of neuroradiological markers, up to the treatment and management of patients in organized pathways. It is a condition with a high prevalence in its chronic manifestations, with predominantly non-acute underpinning neuroimaging markers (3, 4), and with a high incidence in acute manifestations (5), both ischemic and hemorrhagic. These facts highlight SVD as one of the most frequent vascular diseases in aging, a frequent comorbidity in patients presenting with acute cerebrovascular events with other causes, and an important cause of cognitive impairment and functional impairment, due to its gait alteration components.

Certain issues in SVD merit particular attention. Investigation of these can help to increase awareness of the complex interplay of several vascular conditions in aging individuals and those with comorbidities, i.e., the relationship of SVD with classical vascular risk factors and in particular arterial hypertension, its impact on gait, and the correlation between neuroimaging markers and systemic inflammatory biomarkers.

First, arterial hypertension is a strong contributor to SVD and to overt and covert clinical manifestations (6). The relationship between office, ambulatory, and 24-h measurements of blood pressure (BP) (Melgarejo et al.) and neuroimaging markers of SVD (cerebral microbleeds, lacunes, white matter hyperintensities, enlarged perivascular spaces, etc.) is an intriguing topic with relevant implications for the pathophysiology of cerebral microvascular damage and for prevention, also considering the role of sleep and sleeping hours on beta amyloid trafficking/removal in the brain (11). Nighttime BP provides the most valuable prognostic information for adverse health outcomes (12), which can sometimes be attributed to a lack of antihypertensive therapy during the night or to sympathetic modulation of nighttime BP. A set of potential confounding factors is found in the typical comorbidities of hypertensive patients, such as diabetes, dyslipidemia, and obesity. Diabetes is also related to SVD burden through autonomic dysfunction, as suggested by the heart rate variability (HRV) (Qiu et al.) measure. Indeed, HRV is included in risk stratification models in patients with cardiovascular diseases; furthermore, among an inpatient population of diabetic patients, lower HRV has been found to be independently associated with overall burden of SVD. In taking an individual approach to a complex and comorbid patient, it is difficult to identify and tease apart the different and interplaying pathophysiological changes in the cerebral small vessels from hypertension and other vascular risk factors, or to distinguish the roles of concurrent forms of SVD (e.g., type I and type II SVD) (2). It is also challenging to evaluate which BP thresholds should be addressed and targeted for these patients with the objective of slowing the rate of accumulation of SVD burden over a timescale of years and preventing stroke and cognitive impairment (13).

Second, SVD neuroimaging markers are a common finding in aging, and some of these, particularly age-related white matter lesions in the periventricular and deep frontal lobes, are associated with gait and balance impairment (Su et al.) (14). Moreover, white matter lesion load progression is associated with progressive gait impairment even in healthy elderly people. In other studies of older patients with SVD, cerebral microbleeds (CMBs) in the temporal, frontal, and basal ganglia regions have been found to be associated independently of other SVD markers with gait and balance impairment (15). These SVD markers may also be found in patients with other subtypes of stroke (7) and in patients with atrial fibrillation; in a Swiss cohort study, small subcortical infarctions were observed in 368 patients (21%), CMB in 372 (22%), and white matter lesions in 1,715 (99%) (16). SVD burden may contribute to worsening of functional outcomes, while also affecting balance and gait function and increasing the risk of falls. Additionally, the association of white matter hyperintensities with arterial hypertension that also occurs in atrial fibrillation patients (17) is meaningful, and awareness of this is required in order to improve the prevention of cerebrovascular events and cognitive impairment.

Third, the pathophysiology of cerebral damage in SVD is still a matter of debate, involving several mechanisms and triggers with a proposed role for an inflammatory mechanism (Hou et al.) (18). In this regard, systemic inflammation has been proposed as a trigger of a proinflammatory environment in the central nervous system with further acceleration of the molecular cascade involved in SVD, including endothelial dysfunction and breakdown of the blood-brain barrier (19). Among the biomarkers of systemic inflammation, the neutrophil/lymphocyte ratio (NLR) has been linked to cerebrovascular diseases and their prognosis in both ischemic and hemorrhagic stroke. Moreover, NLR is also a potential predictor of the risk of cognitive impairment. The mechanism underlying the association of NLR with cognitive impairment in SVD is still unclear, but inflammation has been implicated as a risk factor for SVD, and immune activation increases the harmful effect of vascular risk factors (20). Another potential issue in the acute evolution of lacunar infarctions is blood rheology, as expressed by viscosity measures (e.g., hematocrit) (Lee et al.). This mechanism contributes to hypoperfusion in small vessels and early neurological deterioration on the clinical side.

SVD is a multifaceted disease, involving several factors that interact in intricate and not always easily discriminable ways. The relevance of SVD in a population setting makes it a highly relevant issue for prevention, treatment, and the organization of health care pathways, both in high-income and in middle-low-income countries. Increased awareness of this condition, together with a coordinated and multinational initiative promoting research and clinical management, is needed in order to overcome the impact of SVD, starting with primary prevention models.

## Author contributions

MZ and RP conceived the paper. MZ wrote the first draft. JL, AK, and RP made substantial contributions and corrected the draft. All authors contributed to the article and approved the submitted version.
